# Supplementary material for: Similar effects of fatigue induced by a repetitive pointing task on local and remote light touch and pain perception in men and women
Source: PLoS One. 2020 Dec 18;15(12):e0244321. doi: 10.1371/journal.pone.0244321 (PMC7748284; doi:10.1371/journal.pone.0244321)
Supplement: Attachment — Submitted filename: ReviewerResponse_Bouffard_PLOSOne2020.pdf [file pone.0244321.s002.pdf]

CRIR Centre de recherche interdisciplinaire en réadaptation du Montréal métropolitain

Hôpital juif de réadaptation
Jewish Rehabilitation Hospital

McGill
Department of Kinesiology and Physical Education
McGill University - Faculty of Education

To the attention of the editor, PLOS One

Object: submission of a revised manuscript: Research article

Quebec, August 16th, 2020

Dear reviewers,

I would first like to thank each of you for the rigorous review made of the submitted manuscript entitled: “Similar effects of fatigue on local and remote sensory and pain perception during a repetitive pointing task in men and women.”. Many important comments were made by each reviewer which help us improve our manuscript. We are please to submit you a revised version of our manuscript in which we addressed most comments. Several changes were made to the manuscript including: a restructuration of the Introduction section, the addition of a Figure (i.e. experimental set-up) and two Tables (i.e. statistical results) and the inclusion of confidence intervals on Figure 4. Some limitations were also discussed in the discussion section as well as a Clinical relevance section. We are confident that the quality and potential impact of the paper were greatly improved, thanks to the insightful comments of the reviewers.

You will find below, answers to each reviewer’s comments along with modifications that were made to the manuscript.

Sincerely,

Jason Bouffard, OT Ph.D.

Table of content

Response to reviewer 1's comments:.. 3
Response to Reviewer 2's comments 10
Response to Reviewer 3's comments 13
Response to Reviewer 4's comments 21

Response to reviewer 1's comments:

Comment	Response
1. My main concern is related to the task. The repetitive pointing task was very well described in your methodology, however, the use of a Borg scale to indicate fatigue and cessation of exercise is very subjective and concerning in my opinion. You cannot guarantee that your subjects, between- and within-sex, exercised at a similar relative volume, which plays an important role on muscle fatigue. Another way to look at this is to measure task-related muscle fatigue; this was considered by the authors but with some flaws (see comment #2 below). Did the authors consider other stoppage criteria? Task failure?	This comment was addressed in the limitation section of the discussion.<br><br>Line 335: As in previous studies, the RPT was stopped when participants reached a RPE ≥ 8/10 [28, 43]. Using another criterion based on, for instance, actual task failure [51] or a predetermined % decrease in maximal strength [52] might have modify the participants' cumulative workload and affect the obtained results. It is, however, important to note that while RPE ratings assess perceived fatigability, performance measures are not as objective as they look. Indeed, performance limitations during a fatiguing task results from the interaction between perceived fatiguability and fatigue-related physiological changes [53]. The relative contribution of subjective and objective phenomena to the impaired performance vary between tasks and individuals. Therefore, it is unlikely that any of the above-mentioned stopping criteria can be considered as a gold standard. Still, it would be interesting to assess the impacts of the stopping criteria on sensory changes induced by a fatiguing task.
2. Another main concern is related to muscle fatigue. Firstly, the authors did not define, or at least did not specify which definition of fatigue they considered while collecting data. It is known that muscular fatigue can be interpreted in many ways. Moreover, the use of EMG to determine fatigue of a muscle is somewhat contradictory. EMG is used to assess electrical activity, which can be high even if the muscle is fatigued. Changes in	We acknowledge that fatigue induced by the repetitive pointing task is multidimensional. The subjective component of fatigue was controlled across participants as it was the stoppage criterion. Regarding the objective component, we only recorded data regarding EMG signs of fatigue, which are frequently used in the literature. Other objective measures of fatigability in the motor domain could have been used. Stimulation techniques at rest or during voluntary contraction, as suggested by

	EMG activity do not represent/indicate muscle fatigue. A proper measure of fatigue would be a baseline vs. post-task muscle force output comparison at a known and constant intensity stimulus, for example, electrical or magnetic stimulation.	Reviewer 1, would provide valuable information. Such technique is however challenging to use during multi-joint tasks. EMG have the advantage of assessing the effects of fatigue induced in several muscles with a relative spatial sensitivity during one or few motor tasks. As in the present study, where we were able to show signs of muscle fatigue in only 2 out of the 4 muscles assessed, others showed evidence of such spatial sensitivity. For instance, Tse et al. 2016 recorded sEMG from 14 muscles acting at the shoulder joint and found that only 4 of them developed signs of fatigue during an arm elevation task. While this limits our secondary objective (relationship between fatigue, PPT and LTT), it does not affect our primary (sex differences in EIH).<br><br>The following information was added to the Introduction (note that the Introduction was restructured in response to a reviewer's comment):<br><br>Line 13: Electromyography (EMG) signs of fatigue, such as a decrease in median frequency associated with an increase in signal amplitude [10], are particularly appealing as they allow an assessment of the relationship between sensory and motor changes during a fatiguing task on a muscle by muscle basis. For instance, Tse et al. 2016 showed the spatial sensitivity of EMG signs of muscle fatigue as they were present in only 4 (i.e. anterior deltoid, posterior deltoid, latissimus dorsi and serratus anterior) out of 14 shoulder muscles assessed during an arm elevation task [11].<br><br>This was added in the limitation section of the Discussion:<br><br>Line 361: A similar limitation can be applied to the fatigue assessment, as it was solely assessed using

	EMG RMS and MDF variables. Other variables could have been chosen from EMG [10] and other signals [56, 57]. While this is a limitation for the assessment of the relationship between fatigue indicators, EIH and LTT, it does not affect the validity of our primary objective, the assessment of sex differences in EIH.
3. The authors did not provide anthropometrical description of their sample. Ideally, strength-matched men and women would provide a better base of comparison. In addition, a simple measurement with a bioimpedance scale (fat and lean mass) would have provided important information to interpret your results.	Agreed, this was added to the limitation section of the discussion.<br><br>Line 353: Furthermore, women in our sample were shorter and (non significantly) lighter than men, as expected [54]. The absolute workload and the linear (but not angular) reaching distance and speed was therefore probably larger for men than for women. Future studies should compare men and women matched for their anthropometric variables to isolate the effects of gender or include these variables as confounding factors given the sample size is sufficient.<br><br>Furthermore, participants' height, weight and body mass index were added in the Methods:<br><br>Line 59: Fifteen men (age = 26.7 ± 6.7 years; height = 176.4 ± 7.1 cm; weight = 73.3 ± 10.8 kg; body mass index =24.6 ± 3.9 kg/m^2) and 15 women (mean age: 26.3 ± 7.3 years; mean height = 168.6 ± 6.8 cm; weight = 67.3 ± 9.0 kg; body mass index =21.8 ± 2.1 kg/m^2) participated in this study. Men and women were significantly different for height ($p < 0.001$) and body mass index ($p = 0.041$), but not for age ($p = 0.857$) and weight ($p = 0.152$).
4. Moreover, it was not indicated whether subjects provided signed consent to participate and if ethics	Agreed, this was added to the Methods section.

were conformed to the Declaration of Helsinki.	Line 69: Ethical approval was received from the Research Ethics Board of the Centre for Interdisciplinary Research in Rehabilitation (CRIR) of Greater Montreal. All participants gave their informewritten consent before their participation to the study and the research was conducted in conformity with the Declaration of Helsinki.
5. As a reader, the way the authors present their results is somewhat hard to follow. For example, it would be easier if important data (such as some of your results and those in your database file) were shown in 1 or 2 tables, pre vs. post task. Moreover, your figures do not have legends as to the different colors used. The use of too much acronyms makes it harder to follow as well. I suggest reviewing which ones are really necessary and removing the others that are not used too often within text.	P values of the Generalized estimation equation were added in Table 1. P values and correlation coefficients were added in Table 2. The numbers were therefore removed from the text as they were all in a Table and in a Figure. We believe that it improved readability.<br><br>Note that the Figures' numbers were changed because of changes in the text and the addition of a Figure 1, showing the experimental set-up.<br><br>Legends regarding the colors used in the Figures were added in the Figure captions.
6. Did the authors consider a mid-/long-term intervention as to simulate a longer fatiguing exposure? In other words, do you think a single and acute exposure to the task in question would elicit musculoskeletal responses similarly to those observed in repetitive task workers? Your rationale was based on this matter, but I do not see how your methodology fit in here (acute vs. long-term exposure).	A section was added in the discussion regarding this point. Moreover, the Introduction was restructured and put less emphasis on the long-term consequences of fatiguing exercises.<br><br>Line 318: **Clinical relevance:**<br><br>The present study is the first, to our knowledge, to assess concurrently muscle fatigue, EIH and non-noxious sensitivity before and after a multi-joint dynamic task. The RPT is more similar to real life activities than single joint isometric or dynamic contractions and its motor consequences have been intensively studied in men and women [22, 28]. Nevertheless, the current study concerns the acute sensory effects of physical activity in healthy individuals. Results

	cannot be extrapolated to people living with musculoskeletal disorders as EIH is often perturbed in such population [14]. Furthermore, the sensory effects of exercise in the long term may be different. On one hand, people performing repetitive upper limb movements in their job are at increased risk of developing musculoskeletal disorders [4]. On another hand, animal and human studies show that regular physical activity can have protective effects against the development of disabling [13]. It is likely that the balance between activity and rest opportunities as well as the characteristics of the physical activity performed influence its effects on sensory functions [5]. Further studies assessing the relationships between an individual's acute responses to physical activities (e.g. EIH) and his or her long-term risk of developing musculoskeletal disorders would be of great interest.<br>.
7. I suggest a careful review on your references; for example, you made reference to a study on Semmes-Weinstein filaments when talking about a cross-correlation function used to remove ECG 'noise' from EMG data (Ref #29, line 169).	References were reviewed
Minor: in some instances you mention 'many' studies and refer to only 1 (lines 56-57); even being a review, it still sounds weird to the reader;	Agreed. In all instances that we notice such formulations, we added the expression (reviewed in [reference]). Furthermore, we added specific references toward the original studies in the following sentences<br><br>Line 253: Still, it should be noted that only two studies reviewed by Naugle et al. (2012) [8] assessed EIH following a dynamic contraction

	[41, 42] and that a recent study showed only modest EIH following dynamic contractions [18].
Minor: line 84: lower limb (“central” ??);	We changed the expression central by remote. Moreover, we used the expression remote when it referred to the site far from the fatigued site thorough the text instead of central or distal.
Minor: line 93: regarding ‘right-handed’, was this an inclusion criteria? If not, please state	This was added to the Methods section:<br><br>Line 66: All participants were right-handed even if this was not an inclusion criterion.
Minor: line 103, you mention PPT and LTT measurements were ‘immediately following’ RPT, my question is how long did it take each muscle to be assessed? Maybe your results would be different if the order of muscles assessment were different? Have the authors considered this?	We agree that the order of the LTT and PPT variable could partly explain why the effects on the former variable appear more consistent than the effects on the latter. This choice was made to avoid contaminating the LTT by applying nociceptive stimuli before it and to be consistent with the testing order in the more comprehensive quantitative sensory testing protocol (Rolke et al. Eur J Pain 2006; 10:77-88).<br><br>However, we do not believe that it is the sole explanation for the weak and localised EIH observed in the present study. The LTT testing in the current study lasted around 2 and 3 minutes. Few studies assessed the duration of EIH following exercise. However, Kosek and Ekholm (Pain. 1995; 61:481-6) showed that pain inhibition was still present 5 minutes following sustained isometric knee extension. Other examples are presented in Koltyn. Sports Med 2000;29: 85-98.<br><br>Moreover, it is important to note that the stimulation sites order was randomized across participants. If the time delay between the motor task and the PPT was the main contributor of the inconsistent EIH, it would have affected all muscles equally.<br><br>The following information was added in the Methods section

	Line: 128: Their dominant arm rested passively on a table with their forearm supinated (palm-up), their elbow extended and their shoulder at 90° of elevation and 40° of horizontal abduction. This posture was chosen as it was close to the position during the RPT. Moreover, as the arm was elevated during the sensory testing, it prevented fatigue recovery by restricting blood flow. Indeed, the recovery of normal sensations is delayed in such position, in comparison to a neutral arm position (unpublished observations).<br><br>[…]<br><br>Line 141: Following LTT measurements (which lasted around 2-3 minutes), PPT measures were taken with an electronic.<br><br>Moreover, the following limitation was added in the discussion.<br><br>Line:365: Finally, in the current study, as in the complete Quantiative sensory testing protocol [58], LTT was always tested before the PPT. We cannot exclude the possibility that increase in LTT were larger and more widespread than the observed EIH because it was tested earlier after the end of the motor task.

Response to Reviewer 2's comments

Comment	Response
1. Can the authors provide a rationale for the lack of kinematic data and why EMG amplitude were not normalized in the current study?<br><br>[…]<br><br>Primarily, the lack of normalization for EMG data	We acknowledge that kinematic data would have allowed us to assess about the relationship between more global motor manifestations of fatigue (i.e. kinematic adaptations) and the sensory changes assessed.<br><br>As for EMG, the lack of amplitude normalization impairs our ability to compare amplitude changes between individuals and between groups. Unfortunately, no kinematic data and no EMG normalization contractions were recorded in this study. Note, however, that the absence of EMG normalization does not preclude the assessment of the changes in EMG frequency content, the most frequently used EMG sign of fatigue in the literature.<br><br>These aspects are already discussed in the limitation section of the Discussion.
2. Is n=15/group sufficient to observe an effect size?	Studies reviewed by Naugle, from which effect size statistics were reported, ranged between 8 and 50 (mean 19.3, standard deviation 8.6). Therefore, our sample size, even when considering groups independently, is comparable to studies available in the literature.<br><br>Regarding the effect size statistics, it is recommended to report it to facilitate between studies comparisons. However, we acknowledge that effect size statistics are more precise for greater sample size. We therefore added the confidence intervals (Figure 4) around the effect size statistic so the reader can acknowledge the precision of our data.<br><br>This was added to the Methods section:

	Line 159: Effect size (ES) statistics with confidence intervals were computed with Hentschket et al.'s *Measures of effect size* Matlab toolbox [36]. Hedges' g were computed for between Sex comparisons. Time effects ES were computed using the mdbysd function as it is a within-subject factor. Confidence intervals were computed using bootstrapping, with 10 000 iterations [37, 38].
3. Given the lack of sex-specific differences, the title is a bit misleading and can be modified to state lack of sex differences.	The title was changed:<br><br>"Similar effects of fatigue on local and distal sensory and pain perception during a repetitive pointing task in men and women"
4. Can the authors provide a rationale for why they chose this study design which could be compared with published findings due to differences in design?	The general study design is consistent with most studies assessing EIH, i.e. assessing pain threshold before and after a fatiguing task. As for the RPT, this task have been used for a decade by our team to study intersegmental adaptations to muscle fatigue.<br><br>These sentences were added to the methods section:<br><br>Line 77: The RPT has been extensively studied for its effects on the motor system and is known to induce EMG and kinematics changes as well as a decrease in maximal force capacity [22, 24, 28, 29], which are all indicators of fatigue. Prior to, and immediately following the RPT, participants underwent PPT and LTT measurements, whereas EMG data was collected during the last 30 seconds of each minute during the RPT. The study design, testing sensory thresholds before and after a fatiguing motor task is

	consistent with previous studies evaluating sex differences in EIH (e.g. [19, 21]).

Response to Reviewer 3's comments

Comment	Response
1. Primarily, the lack of normalization for EMG data	The lack of amplitude normalization impairs our ability to compare amplitude changes between individuals and between groups. Unfortunately, no kinematic data and no EMG normalization contractions were recorded in this study. Note, however, that the absence of EMG normalization does not preclude the assessment of the changes in EMG frequency content, the most frequently used EMG sign of fatigue in the literature.<br><br>These aspects are already discussed in the limitation section of the Discussion.
2. no disclosure of further participant characteristic data (i.e., weight, height, lever arm length, activity history)<br><br>[…]<br><br>Additionally, please separate the average ages for males and females.<br><br>[…]<br><br>Please include more descriptive participant characteristics for each sex; i.e., weight, height, forearm/arm length, general activity history (were these moderately active individuals, athletes, sedentary, or a mixed group? Were individuals screened for a history of resistance/weight training?).	Participants were all recruited by the research team through their social network, which generally include young and physically active individuals. However, physical activity history was not assessed formally. Participants' height, weight and body mass index were added.<br><br>This was added to the Methods section:<br><br>Line 59: Fifteen men (age = 26.7 ± 6.7 years; height = 176.4 ± 7.1 cm; weight = 73.3 ± 10.8 kg; body mass index =24.6 ± 3.9 kg/m^2) and 15 women (mean age: 26.3 ± 7.3 years; mean height = 168.6 ± 6.8 cm; weight = 67.3 ± 9.0 kg; body mass index =21.8 ± 2.1 kg/m^2) participated in this study. Men and women were significantly different for height ($p < 0.001$) and body mass index ($p = 0.041$), but not for age ($p = 0.857$) and weight ($p = 0.152$).
3. no randomization of timing for testing pressure pain threshold (PPT) before the light touch threshold (LTT; LTT was	We agree that the order of the LTT and PPT variable could partly explain why the effects on the former variable

assessed before PPT in all cases, potentially leading to null findings given a possible quick recovery time for measures of interest),<br><br>[…]<br><br>If PPT was assessed after LTT for all participants post-fatigue, is it possible that the amount of time between cessation of the protocol to the beginning of assessing PPT was enough to allow for any potential effect of sex to recover? If you had tested this measure immediately following fatigue instead of testing LTT first, perhaps there would be a different story. Can you speculate on this?<br><br>[…]<br><br>- Line 323-324: Again, it may be possible that these effects were different based on how time-sensitive they are to when the fatiguing task ceased. In that case, a larger effect should be expected for LTT.<br><br>[…]<br><br>Some of these concerns are with the design of the study (ie. Not testing PPT before LTT, not randomizing these measures, and/or not having two separate testing days to account for differences in time-from-fatigue),	appear more consistent than the effects on the latter. This choice was made to avoid contaminating the LTT by applying nociceptive stimuli before it and to be consistent with the testing order in the more comprehensive quantitative sensory testing protocol (Rolke et al. Eur J Pain 2006; 10:77-88).<br><br>However, we do not believe that it is the sole explanation for the weak and localised EIH observed in the present study. The LTT testing in the current study lasted around 2 and 3 minutes. Few studies assessed the duration of EIH following exercise. However, Kosek and Ekholm (Pain. 1995; 61:481-6) showed that pain inhibition was still present 5 minutes following sustained isometric knee extension. Other examples are presented in Koltyn. Sports Med 2000;29: 85-98.<br><br>Moreover, it is important to note that the stimulation sites order was randomized across participants. If the time delay between the motor task and the PPT was the main contributor of the inconsistent EIH, it would have affected all muscles equally.<br><br>The following information was added in the Methods section<br><br>Line: 128: Their dominant arm rested passively on a table with their forearm supinated (palm-up), their elbow extended and their shoulder at 90° of elevation and 40° of horizontal abduction. This posture was chosen as it was close to the position during the RPT. Moreover, as the arm was elevated during the sensory testing, it prevented fatigue recovery by restricting blood flow. Indeed, the recovery of normal sensations is delayed in

	such position, in comparison to a neutral arm position (unpublished observations).<br><br>[…]<br><br>Line 141: Following LTT measurements (which lasted around 2-3 minutes), PPT measures were taken with an electronic.<br><br>Moreover, the following limitation was added in the discussion.<br><br>Line:365: Finally, in the current study, as in the complete Quantiative sensory testing protocol [58], LTT was always tested before the PPT. We cannot exclude the possibility that increase in LTT were larger and more widespread than the observed EIH because it was tested earlier after the end of the motor task.
4. no disclosure of any metronome adjustments for task frequency based on arm length to account for differences in distance traveled.<br><br>[…]<br><br>Was the timing of the metronome adjusted for participants with longer arms, given they would have had to travel a longer distance between targets? If not, what was the rationale for keeping these the same? If you took measures of arm length, you can assess for a sex difference to see if perhaps one sex had to travel greater distances than another sex.	The metronome was not adjusted to account for differences in arm length and travel distance. Indeed, while the linear displacements changes according to the participant's arm length, the angular excursions (therefore its velocity) should similar across participants. This is supported by kinematics data collected in previous studies during the same task showing similar range of motion at the elbow and shoulder between men and women (Bouffard et a. Biol Sex Differ. 2018; 9:17).. Moreover, in the same article, we reported the reaching errors (distance between the finger and the target at the end of each movement), which was the same between men and women. This suggest that the task was of similar difficulty despite the longer travel distance for men, who are on average taller.

5. Introduction: The general flow of the introduction is present, but the transition between concepts is choppy. This makes it difficult to follow the storyline projected by the authors such that a strong defence for their hypotheses might be presented. The authors highlight the importance of investigating sex-related differences in nsMSD in the first paragraph and follow this in the next paragraph by a discussion of the link between fatigue, pain, and MSD. The main objective highlights the desire to assess light touch, pain pressure thresholds, and EMG measures of fatigue; however, the former two are not clearly differentiated in the introduction. This should be addressed.	The Introduction section was restructured to improve concision and readability.
6. Additionally, why was it important to differentiate between local vs distal effects; that is, why would it be assumed that LTT and PPT would have distal effects yet EMG measures of fatigue would not? Please highlight this briefly in the introduction.	EIH experienced at the site of exercise may be related to a combination of local and more widespread, central phenomena. As for EMG signs of fatigue, they are more related to local effects, especially for the median frequency changes. EMG signs of fatigue is therefore useful to assess the relationships between EIH occurring at a given segment with localized changes that occurs because of fatigue. This related information was added in the Introduction section.<br><br>Line 13: Electromyography (EMG) signs of fatigue, such as a decrease in median frequency associated with an increase in signal amplitude [10], are particularly appealing as they allow an assessment of the relationship between sensory and motor changes during a fatiguing task on a muscle by muscle basis. For instance, Tse et al. 2016 showed the spatial sensitivity of EMG signs of muscle fatigue as they were present in only 4 (i.e. anterior deltoid, posterior

	deltoid, latissimus dorsi and serratus anterior) out of 14 shoulder muscles assessed during an arm elevation task [11].
7. - Line 58-60: Stating “EIH” followed by “induced hypoalgesia” comes across as repetitive. Consider rewording to improve clarity.	This was fixed
8. - Line 62: The authors mentioned a number of neurotransmitters and that these contribute to EIH – do they all enhance effects of EIH or do some neurotransmitters work to oppose it? Please include the directionality of their contribution.	We modified this sentence. Note that the Introduction was restructured according to another comment.<br><br>Line 20: Although the mechanisms of EIH remain unclear, pain inhibitory opioid and non-opioid mechanisms appear to be involved as naloxone (an opioid receptors antagonist) decrease, but only partly and inconsistently, hypoalgesia (reviewed in [12]). Other biological (e.g. serotonergic, immune and autonomic nervous system) and psychosocial factors can also interact together and affect the effects of exercises on pain sensitivity, either positively or negatively (e.g. balance between proinflammatory and anti-inflammatory interleukins) [13, 14].
9. - Line 93: Please indicate whether this is SD or SEM for the participant ages..	This was added in the statistical analyses section:<br><br>Standard deviations are presented in the text.
10. - Were all participants screened for usage of antidepressant/anxiolytic medications? Had any taken pain medications prior to testing?	Participants filled the Par-Q questionnaire to be included in the study. The use of such medications would have been identified in the following questions:

	Have you ever been diagnosed with another chronic medical condition (other than heart disease or high blood pressure)?<br><br>Are you currently taking prescribed medications for a chronic medical condition?<br><br>Do you currently have (or have had within the past 12 months) a bone, joint, or soft tissue (muscle, ligament, or tendon) problem that could be made worse by becoming more physically
11. - Were female participants screened for contraceptive medication usage and/or tested at a certain point during their menstrual cycle? if not, please highlight the rationale for this decision in the manuscript.	Women were not screened for contraceptive medication or for their menstrual cycle.<br><br>This was added in the methods section:<br><br>Line 67: Women were not screened for contraceptive medication and were not tested at a certain point during their menstrual cycle. This decision was justified given the inconclusive evidence regarding such factors on fatigability and on EIH [26, 27].
12. - Indicate whether all participants gave written informed consent prior to starting the experimental protocol.	Agreed, this was added to the Methods section.<br><br>Line 69: Ethical approval was received from the Research Ethics Board of the Centre for Interdisciplinary Research in Rehabilitation (CRIR) of Greater Montreal. All participants gave their informewritten consent before their participation to the study and the research was conducted in conformity with the Declaration of Helsinki.

13. - Lines 110-111: Was stance regulated for participants with different heights? If so, how.	Participants were free to adopt a comfortable stance at the beginning of the experiment. It is likely that this natural stance was influenced by participant's anthropometry.<br><br>This was added in the Methods<br><br>Line: 88 Throughout the task, their feet remained placed on a zone marked on the floor with tape, which was adjusted for each participant's comfortable standing position.
14. - Line 130-131: How often was RPE assessed by the experimenter? Was this assessment consistent throughout the protocol and across participants	Participants were asked to report the RPE every 30 seconds (line 106).
15. - Line 137: Please indicate the diameter of the recording surface of the electrodes.	This information was added in the Methods<br><br>Line 116: Immediately after the pre-fatigue LTT and PPT measurements, but before the RPT, pre-gelled bipolar Ag-AgCl surface electrodes (Ambu, Denmark; 1 cm diameter; 3 cm interelectrode distance) were placed over the following sites according to SENIAM recommandations:
16. - Line 170-171: Why did was the backward arm motion not assessed?	Only forward motions were analyzed for concision. Note that conclusions are globally similar when backward motions are analyzed.
17. - What statistical software was used for your assessments?	This was added<br><br>Line 169: Statistical analyses were computed with SPSS 23 (IBM, USA) and Matlab 2019b (The Mathworks, USA).
18. - Please indicate your pre-determined p-value for setting statistical significance.	This was added<br><br>Line 170: The level of significance of statistical analyses was set to $p \leq 0.05$.

19. - Line 221: Please remove the word "trends" as there is no statistical significance for the values presented.	This was fixed<br><br>Line 194:No significant time effects were observed for the UT […]
20. - Line 316: should read "previous study" not "previous research"	This was fixed
21. - Line 339: This is the first time the term "whole-body dynamic task" has shown up in reference to the repetitive pointing task. This task cannot be considered whole-body given that it primarily works the upper limb and has no involvement from the lower limb, other than what is required for quiet standing, and therefore is not comparable to true whole-body dynamic exercise such as running or cycling. Please revise the terminology.<br><br>[…]<br><br>Line 354: Please see note above about the term "whole-body dynamic task"	This was fixed<br><br>Line 319: The present study is the first, to our knowledge, to assess concurrently muscle fatigue, EIH and non-noxious sensitivity before and after a multi-joint dynamic task.<br><br>[…]<br><br>Line 371: This study extends the literature on sex-specific exercise induced hypoalgesia (EIH) by using a multi-joint dynamic task designed to mimic occupational tasks.

Response to Reviewer 4's comments

Comment	Response
1. . the authors have used RMS and MDF of EMG for assessment of the muscle fatigue. However, recent works use different measures [RCIM19]. The authors should mention novel measures, and they should motivate the reasons why they have used EMG RMS and MDF. For istance, why joint torque is not included?<br><br>[RCIM19] Peternel, Luka, et al. "A selective muscle fatigue management approach to ergonomic human-robot co-manipulation." Robotics and Computer-Integrated Manufacturing 58 (2019): 69-79.	We acknowledge that fatigue induced by the repetitive pointing task is multidimensional. The subjective component of fatigue was controlled across participants as it was the stoppage criterion. Regarding the objective component, we only recorded data regarding EMG signs of fatigue, which are frequently used in the literature. Other objective measures of fatigability in the motor domain could have been used. EMG have the advantage of assessing the effects of fatigue induced in several muscles with a relative spatial sensitivity during one or few motor tasks. As in the present study, where we were able to show signs of muscle fatigue in only 2 out of the 4 muscles assessed, others showed evidence of such spatial sensitivity. For instance, Tse et al. 2016 recorded sEMG from 14 muscles acting at the shoulder joint and found that only 4 of them developed signs of fatigue during an arm elevation task. While this limits our secondary objective (relationship between fatigue, PPT and LTT), it does not affect our primary (sex differences in EIH).<br><br>The following information was added to the Introduction (note that the Introduction was restructured in response to a reviewer's comment):<br><br>Line 13: Electromyography (EMG) signs of fatigue, such as a decrease in median frequency associated with an increase in signal amplitude [10], are particularly appealing as they allow an assessment of the relationship between sensory and motor changes during a fatiguing task on a muscle by muscle basis. For

	instance, Tse et al. 2016 showed the spatial sensitivity of EMG signs of muscle fatigue as they were present in only 4 (i.e. anterior deltoid, posterior deltoid, latissimus dorsi and serratus anterior) out of 14 shoulder muscles assessed during an arm elevation task [11].<br><br>This was added in the limitation section of the Discussion:<br><br>Line 361: A similar limitation can be applied to the fatigue assessment, as it was solely assessed using EMG RMS and MDF variables. Other variables could have been chosen from EMG [10] and other signals [56, 57]. While this is a limitation for the assessment of the relationship between fatigue indicators, EIH and LTT, it does not affect the validity of our primary objective, the assessment of sex differences in EIH.
2. the authors should include some pictures of the people performing the task with the EMG placement on the body.	A picture of the experimental set up was added. However, note that the picture was taken from another project studying the same motor task. Therefore, reflective markers and electrodes over the pectoralis major muscles are apparent despite the fact that they were not collected in the current experiment. Limited access to the lab given the Covid 19 pandemic prevent us from taking a new picture. This Figure caption was added.<br><br>Line 104: **Fig 1. Experimental set-up for the repetitive pointing task.** Note that in the present study, kinematics and

	pectoralis major EMG data were not collected.
3. the authors have performed a statistical analysis using the effect size. They should include the hypotheses on the distribution of data to evaluate the effect size (normality and homogeneity of variance). However, does it make sense to perform the effect size analysis if there is not a significative differences in the data (line 200)?	Normality and homogeneity of variance are not reported as inferential statistics were done with Generalized estimation equation.<br><br>As for effect size statistics, they can be applied for even for non-normal data. However, in such cases, it is preferable to compute confidence intervals using bootstrapping rather than parametric methods, which was done in the revised version of the manuscript.<br><br>Homogeneity of variance was respected in most of the assessed comparisons (11 on 16 variables for both Sex and Time comparisons). However, data were not normally distributed for the majority of variables.<br><br>Still, in the published literature on sex differences on exercise induced hypoalgesia, effect size statistics similar to those used in the present article are reported. We therefore believe that these are preferable to compare our results with previously published studies.<br><br>Furthermore, we believe that effect size statistics should be reported even for non significant statistical differences. As effect size statistics are not influenced by sample size, reporting them allow the reader to judge whether non-significant differences are caused by a real lack of (or small) effects or by an insufficient sample size.<br><br>Changes in the manuscript:<br><br>confidence intervals on Figure 4.

	Line 159: Effect size (ES) statistics with confidence intervals were computed with Hentschket et al.'s *Measures of effect size* Matlab toolbox [36]. Hedges' g were computed for between Sex comparisons. Time effects ES were computed using the mdbysd function as it is a within-subject factor. Confidence intervals were computed using bootstrapping, with 10 000 iterations [37, 38].
4. the author should include the significance level of the p-value. Without it, it is difficult to give an interpretation on the p-values equal to 0.096 and 0.071 (line 222)	Table 1 and Table 2 were added to present results of generalized estimation equations and correlation analyses, respectively.
5. based on the achieved results, the authors should include a discussion on possible future works related to understanding of the real causes of the WMSD in the women	A section was added in the discussion regarding this clinical relevance of the presented work. Moreover, the Introduction was restructured and put less emphasis on the long-term consequences of fatiguing exercise.<br><br>**Line 318: Clinical relevance:**<br><br>The present study is the first, to our knowledge, to assess concurrently muscle fatigue, EIH and non-noxious sensitivity before and after a multi-joint dynamic task. The RPT is more similar to real life activities than single joint isometric or dynamic contractions and its motor consequences have been intensively studied in men and women [22, 28]. Nevertheless, the current study concerns the acute sensory effects of physical activity in healthy individuals. Results cannot be extrapolated to people living with musculoskeletal disorders as EIH is often perturbed in such population [14]. Furthermore,

	the sensory effects of exercise in the long term may be different. On one hand, people performing repetitive upper limb movements in their job are at increased risk of developing musculoskeletal disorders [4]. On another hand, animal and human studies show that regular physical activity can have protective effects against the development of disabling [13]. It is likely that the balance between activity and rest opportunities as well as the characteristics of the physical activity performed influence its effects on sensory functions [5]. Further studies assessing the relationships between an individual's acute responses to physical activities (e.g. EIH) and his or her long-term risk of developing musculoskeletal disorders would be of great interest.
