# Supplementary material for: Similar effects of fatigue induced by a repetitive pointing task on local and remote light touch and pain perception in men and women
Source: PLoS One. 2020 Dec 18;15(12):e0244321. doi: 10.1371/journal.pone.0244321 (PMC7748284; doi:10.1371/journal.pone.0244321)
Supplement: Attachment — Submitted filename: Bouffardetal2020_PLOSone_Responsetoreviewer.pdf [file pone.0244321.s003.pdf]

CRIR
Centre de recherche interdisciplinaire en réadaptation du Montréal métropolitain

Hôpital juif de réadaptation
Jewish Rehabilitation Hospital

McGill
Department of Kinesiology and Physical Education
McGill University - Faculty of Education

To the attention of the reviewers, PLOS One

Object: submission of a revised manuscript: Research article

Quebec City, November 20th, 2020

Dear reviewers,

I would first like to thank you for the high quality comments made in the current and previous review rounds for our manuscript entitled: "Similar effects of fatigue induced by a repetitive pointing task on local and remote light touch and pain perception in men and women". We are pleased to submit you a revised version of our manuscript in which we addressed the remaining comments you formulated. The manuscript was entirely reviewed to correct grammatical errors as highlighted by reviewer 3. Some information was also added in the Limitations section of the discussion.

You will find answers to each reviewer's comments along with modifications that were made to the manuscript below.

Sincerely,

Jason Bouffard, OT Ph.D.

Rev. #	Comment	Response	Lines changed in the manuscript
1	I still think the use of RPE scores as stoppage criterion is a limitation and a flaw in your design. I agree with your response but I still believe a better approach to this would be matching the cumulative work performed by your male and female subjects.<br><br>This should be listed as a limitation.	The following changes were made in the limitations section:<br><br>**Limitations:**<br><br>As in previous studies, the RPT was stopped when participants reached a RPE ≥ 8/10 [28, 43], which may represent a limitation of the study design.<br><br>[…]<br><br>A more straightforward approach would be to use a fixed motor task duration to ensure that the cumulative work performed by men and women is the same.	p.18, line 333
	2: I agree it is difficult to assess fatigue on a multi-joint task, involving multiple muscles. As you mentioned, EMG is a good way to infer signs of fatigue, however, I still believe it has its limitations and you could have used another way to assess fatigue. Possibly, of all muscles tested, you could have picked one that is the most 'fatigable' in the literature, and tested for fatigue in the way I mention (baseline vs post-task, force output after a non-volitional contraction - electrical/magnetic stimulus).	This was added in the limitations section:<br><br>A similar limitation can be applied to the fatigue assessment, as it was solely assessed using EMG RMS and MDF variables. Other variables could have been chosen from EMG [10] and other signals [56, 57]. Future studies could also use electrically induced muscle contractions, triggered at rest or superimposed to a MVC, to assess fatigue in the muscle and central	p. 19, line 363

		nervous system.in the muscle and the central nervous system.	
2	2: I have no further comments. The authors have addressed all concerns that were raised in the previous round and provided new data as well as analysis.	No further changes made.	
3	The authors have adequately addressed my main comments. My only major remaining concern are the grammatical errors throughout the manuscript (syntax, etc) that will impair the ability of a reader to understand certain statements. The authors should have the manuscript thoroughly reviewed by a native English speaker with an academic writing background.	The manuscript was carefully reviewed for grammatical errors. Minor changes were made accordingly.	
	L12: "shorter duration contractions" - do you mean high intensity contractions with a short duration? This sentence reads as though these shorter duration contractions are at a low/moderate contraction intensity, which would imply that the resulting fatigue should be less than the long duration contractions. Revise accordingly.	This was corrected.<br><br>Muscle fatigue may play a crucial role in EIH, as low to moderate intensity contractions maintained until exhaustion appear to be more effective at inhibiting pain than stronger, shorter duration, contractions [8, 9].	p.4, line 10
	L20-25: Grammatical errors within this sentence make it very difficult to navigate. Please revise. I have refrained from making further comments about language, as I believe this paper would greatly benefit from professional editorial services.	This was corrected:<br><br>Although the mechanisms of EIH remain unclear, both opioid and non-opioid pain inhibitory systems appear to be involved. Indeed, naloxone (an opioid receptor antagonist) decreases only partly and inconsistently hypoalgesia (reviewed in [12]).	p.4, line 21
	Methodology: The authors noted in their review response that participants were not screened for physical activity history. I would strongly encourage future studies incorporate at least a basic measure of physical activity screening. Resistance training history or professional sports (i.e., baseball, rugby, basketball) can heavily influence fatiguability of upper limb muscles.	This was added in the limitations section<br><br>Future studies should compare men and women matched for their anthropometric variables, in order to isolate specific mechanisms underlying the effects of sex, or include these variables as	p.19, line 356

		confounding factors given the sample size is sufficient. Other personal characteristics (e.g. fitness, history of resistance training and sports participation) should also be considered.	
